# Expression of the Nonclassical MHC Class I, Saha-UD in the Transmissible Cancer Devil Facial Tumour Disease (DFTD)

**DOI:** 10.3390/pathogens11030351

**Published:** 2022-03-14

**Authors:** Kathryn Hussey, Alison Caldwell, Alexandre Kreiss, Karsten Skjødt, Annalisa Gastaldello, Ruth Pye, Rodrigo Hamede, Gregory M. Woods, Hannah V. Siddle

**Affiliations:** 1 School of Biological Sciences, University of Southampton, Southampton SO17 1BJ, UK; k.a.hussey@soton.ac.uk (K.H.); aly.caldwell@googlemail.com (A.C.); annalisa80it@yahoo.it (A.G.); 2 Menzies Institute for Medical Research, University of Tasmania, Hobart, TAS 7000, Australia; alexandre.kreiss@utas.edu.au (A.K.); ruth.pye@utas.edu.au (R.P.); g.m.woods@utas.edu.au (G.M.W.); 3 Department of Cancer and Inflammation, University of Southern Denmark, 5230 Odense, Denmark; kskjoedt@health.sdu.dk; 4 School of Biological Sciences, University of Tasmania, Hobart, TAS 7005, Australia; rodrigo.hamedeross@utas.edu.au

**Keywords:** transmissible cancer, Tasmanian devils, MHC class I, nonclassical, immune evasion

## Abstract

Devil facial tumour disease (DFTD) is a transmissible cancer that has circulated in the Tasmanian devil population for >25 years. Like other contagious cancers in dogs and devils, the way DFTD escapes the immune response of its host is a central question to understanding this disease. DFTD has a low major histocompatibility complex class I (MHC-I) expression due to epigenetic modifications, preventing host immune recognition of mismatched MHC-I molecules by T cells. However, the total MHC-I loss should result in natural killer (NK) cell activation due to the ‘missing self’. Here, we have investigated the expression of the nonclassical MHC-I, Saha-UD as a potential regulatory or suppressive mechanism for DFTD. A monoclonal antibody was generated against the devil Saha-UD that binds recombinant Saha-UD by Western blot, with limited crossreactivity to the classical MHC-I, Saha-UC and nonclassical Saha-UK. Using this antibody, we confirmed the expression of Saha-UD in 13 DFTD tumours by immunohistochemistry (n = 15) and demonstrated that Saha-UD expression is heterogeneous, with 12 tumours showing intratumour heterogeneity. Immunohistochemical staining for the Saha-UD showed distinct patterns of expression when compared with classical MHC-I molecules. The nonclassical Saha-UD expression by DFTD tumours in vivo may be a mechanism for immunosuppression, and further work is ongoing to characterise its ligand on immune cells.

## 1. Introduction

Devil facial tumour disease (DFTD) is a transmissible cancer circulating in the Tasmanian devil (*Sarcophilus harrisii*), a marsupial species endemic to the Australian island of Tasmania [1]. These tumour cells transmit as an allograft between devils due to biting behaviour during feeding and mating [2,3,4], forming new tumours on the face and/or neck of the new host. The disease was first observed in 1996 [5] and has since spread across most of the island, leading to a population decline [6] and the registration of Tasmanian devils as an endangered species in 2008 [7]. However, recent phylogenetic analysis of DFTD indicates the cancer is becoming endemic, with reduced transmission rates over time [8], along with an increase in tolerance [9,10,11,12].

Transmitting as an allograft, DFTD should elicit an immune response due to the disparate major histocompatibility complex class I (MHC-I) molecules between the cancer and its host. MHC-I molecules consist of a heavy chain and a β_2_-microglobulin (β_2_m) molecule, which bind to intracellular self or nonself peptides. Classical MHC-I molecules are expressed on the surface of most cells and present peptides to CD8^+^ cytotoxic T cells via the T cell receptor [13,14]. An immune response can be activated if the T cell recognises a nonself or mutated self peptide (for example, from a virus or cancer cell, respectively) or a nonself MHC-I heavy chain, such as in a transplant. Though devils have low genetic diversity at the MHC-I loci [15,16], it has been shown that devils are able to reject both MHC-I-matched and mismatched skin grafts [17], yet an immune response to DFTD is rarely observed. Six wild devils have been found to produce serum antibodies against DFTD, four of these associated with regressions [18], but these cases are rare, with most devils appearing ignorant of a DFTD infection. Interestingly, some devils are able to tolerate infection, with increased survival in females associated with the host SNPs in genes linked to cell-cycle regulation, cell adhesion, and immunity, though no link was found in males [11].

An important mechanism of immune evasion in DFTD is the loss of MHC-I expression through epigenetic modifications of genes that encode components of the antigen presentation pathway, β_2_m and TAP (transporter associated with antigen processing) [19]. These genes can be upregulated by treatment with the inflammatory cytokine interferon-gamma (IFN-γ) [19] or overexpression of transcriptional coactivator NLRC5 [20]. More recently, it has been found that DFTD expresses polycomb repressive complex 2 (PRC2), which is a conserved mechanism for transcriptional silencing of the antigen presentation pathway in tumours with extremely low MHC-I expression [21].

Low levels of classical MHC-I at the cell surface should trigger a natural killer (NK) cell response due to a lack of MHC-I expression, termed ‘missing self’ [22]. While devil NK cell markers are not well defined, making study of these cells difficult, it has been found that devils have functional NK cells that can engage in the cytotoxic killing of the human cancer cell line, K562, which lacks MHC-I on the cell surface; though this response was not seen against DFTD cells [23]. As a result, it is expected that DFTD cells are employing additional mechanisms to evade the host immune system.

There are two broad categories of MHC-I molecules: classical and nonclassical. Classical MHC-I are highly polymorphic, expressed by most cell types, and bind intracellular peptides for presentation to T cells at the cell surface [24,25]. Nonclassical MHC-I genes have low levels of polymorphism and can bind a variety of ligands, including peptides and lipids. These molecules can interact with a broader range of immune cells, including, but not limited to, NK cells [24,25,26] where they have an established role as inhibitory ligands [26].

In the Tasmanian devil, there are three classical genes: *Saha-UA*, *Saha-UB*, and *Saha-UC* [16]; in addition, there are six nonclassical genes: *Saha-UD*, *Saha-UK* [16], *Saha-UM*, *Saha-CD1*, *Saha-MR1* [27], and *Saha-UT* [28]. Eleven *Saha-UD* alleles have been identified (at the amino acid level), and all have high nucleotide sequence similarity (>97.7%) with putative peptide binding residues not under positive selection [27]. Amino acid differences between the alleles are low, and few of these residues are predicted to interact with peptides or the T cell receptor [29]. *Saha-UD*, while it is in a separate phylogenetic clade [16], is closely related to the classical MHC-I genes [27] and binds to β_2_m [30]; therefore, it is hypothesised that Saha-UD molecules bind peptides for antigen presentation at the cell surface. *Saha-UK* is monomorphic [27]; therefore, its expression would not be expected to activate an immune response. However, it is possible it does not bind to β_2_m [30] and is predicted to have a marsupial-specific function [16]. Due to the expression of Saha-UD in DFTD cell lines, its potential for peptide presentation, and its low polymorphism in the population, we investigated the expression of nonclassical Saha-UD in DFTD primary tumours as a mechanism for host immune evasion.

## 2. Results

### 2.1. Anti-Saha-UD Antibody, α-14-37-3, Specifically Binds Recombinant Nonclassical Saha-UD Protein

*Saha-UD* is a devil MHC-I gene that has been classified as nonclassical due to low polymorphisms between alleles and tissue-specific expression [16]. However, phylogenetic analysis groups *Saha-UD* with the classical MHC-I genes, *Saha-UA*, *Saha-UB*, and *Saha-UC* [16,27,31]; while other nonclassical MHC-I genes, *Saha-UK* and *Saha-UM*, form a separate clade, and *Saha-CD1* and *Saha-MR1* group with orthologues in other species [27]. Sequence identity analysis on the alpha 1–alpha 2 domain amino acid sequence of the devil classical MHC-I (SahaI-01) and nonclassical Saha-UD (SahaI*12), Saha-UK, and Saha-UM is shown in Table 1, confirming sequence identity is highest between classical MHC-I and the nonclassical Saha-UD at 78.2%. The sequence identity and phylogenetic analysis indicates that while Saha-UD shares evolutionary history with the devil classical MHC-I, its polymorphism and expression may denote a unique function.

**Figure 1:**
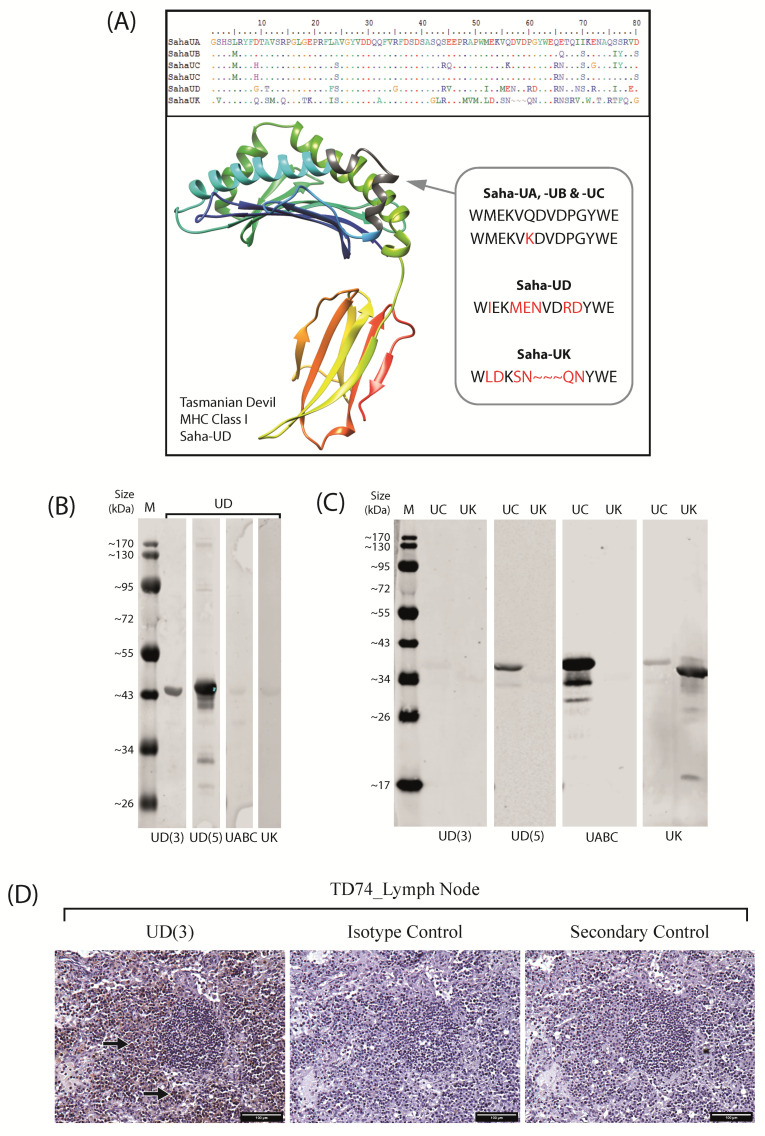
**Nonclassical MHC class I, Saha-UD antibody generation and validation.** (**A**) Antibodies were generated against the peptide sequence ‘WIEKMENVDRDYWE’ from the alpha-1 domain of the nonclassical MHC class I, Saha-UD heavy chain protein. This sequence differs from the classical MHC class I (Saha-UA, -UB, and -UC) and nonclassical Saha-UK sequences, shown in the amino acid alignment. A putative model of the Saha-UD molecule is shown, with the antibody epitope in grey. In the grey box, red indicates where the sequences differ. An antibody against the Saha-UA-UB-UC was generated previously, using the sequence ‘WMEKVQDVDPGYWE’ from the alpha-1 domain [32]. (**B**,**C**) Two anti-Saha-UD antibodies, clones α-UD_14-37-3 (UD(3)) and α-UD_14-37-5 (UD(5)), were screened by Western blot for protein specificity. Blots were stained with antibodies against the classical Saha-UA, -UB, and -UC (UABC), α-UA/UB/UC_15-25-8, and nonclassical Saha-UK (UK), α-UK_15-29-1, as positive controls for their respective proteins and negative controls for other MHC class I. Blots were run with the following: (**B**) recombinant Saha-UD heavy chain protein; (**C**) recombinant Saha-UC and Saha-UK heavy chain proteins. (**D**) Formalin-fixed paraffin-embedded Tasmanian devil submandibular lymph node samples were stained by immunohistochemistry with the α-UD_14-37-3 (UD(3)) anti-Saha-UD antibody, along with a secondary antibody only and an IgG2a isotype antibody as controls. Images were taken at 20× magnification. Positive cells are stained brown; nuclei are stained blue. Black arrows indicate cells with positive staining. Scale bars = 100 µm.

To investigate Saha-UD expression further, anti-Saha-UD antibodies were generated by immunising mice using the peptide sequence ‘WIEKMENVDRDYWE’. This sequence comes from the same region of the alpha-1 domain as has been used previously to generate anti-Saha-UA-UB-UC antibodies [32] and has low sequence similarity to the classical MHC-I sequences and nonclassical MHC-I, Saha-UK (Figure 1A).

Eight anti-Saha-UD antibody clones were screened by immunohistochemistry (IHC) using formalin-fixed paraffin-embedded (FFPE) devil spleen samples (Figure S1). From this data, two antibodies were chosen that gave the strongest staining with minimal background: clone α-UD_14-37-3 (hereafter referred to as UD(3)) and α-UD_14-37-5 (UD(5)). The recombinant Saha-UD heavy chain protein was stained with the UD(3) and UD(5) antibodies via Western blot, along with a pan-specific anti-Saha-UA-UB-UC antibody (α-UA/UB/UC_15-25-8) and an anti-Saha-UK antibody (α-UK_15-29-1) [32], hereafter referred to as UABC and UK antibodies, respectively, as negative controls (Figure 1B). UD(3) and UD(5) both identified the Saha-UD recombinant protein, giving a band at ~44 kDa, the size expected for the recombinant protein. UD(5) produced stronger staining but also stained for multiple bands above and below the ~44 kDa band for the Saha-UD, which were not seen on the UD(3) blot. The UABC and UK antibodies both gave a very faint band for the Saha-UD, validating that these antibodies do not have a strong affinity for the nonclassical Saha-UD.

Recombinant MHC-I heavy chain proteins, classical Saha-UC, and nonclassical Saha-UK were probed with the anti-Saha-UD antibodies to exclude the possibility of cross-reactivity with the closely related devil MHC-I heavy chain proteins (Figure 1C). As expected, the UABC and UK antibodies stained, respectively, for the Saha-UC (~38 kDa) and Saha-UK (~35 kDa) recombinant proteins, with the UK antibody also binding weakly to the Saha-UC protein. For their respective proteins, there were additional bands at a lower molecular weight. As these were purified recombinant protein samples, these bands may be due to protein degradation or alternative splicing. This was tested by running the proteins on an SDS-PAGE gel and staining with Coomassie Blue, where the same bands were observed (Figure S2). The UD(3) antibody produced very faint bands for the Saha-UC and Saha-UK recombinant proteins. As devil classical MHC-I molecules have a high sequence similarity [31], the result observed for the Saha-UC is expected to reflect all classical MHC-I. Therefore, these results indicate the UD(3) antibody does not have a strong binding affinity for either the classical MHC-I or nonclassical Saha-UK and is specific for the nonclassical Saha-UD. The UD(5) antibody did not produce a band for the Saha-UK protein; however, it did stain for the Saha-UC (~38 kDa). Therefore, it is not a specific antibody against the nonclassical MHC-I, Saha-UD (Figure 1C). To further validate the specificity of the UD(3) antibody, serial lymph node samples were stained using a mouse IgG2a isotype control antibody and a secondary-only control (Figure 1D).

### 2.2. Nonclassical Saha-UD Is Expressed in Primary DFTD Tumours, but Expression Varies and Tumours Show Intratumour Heterogeneity

**Figure 2:**
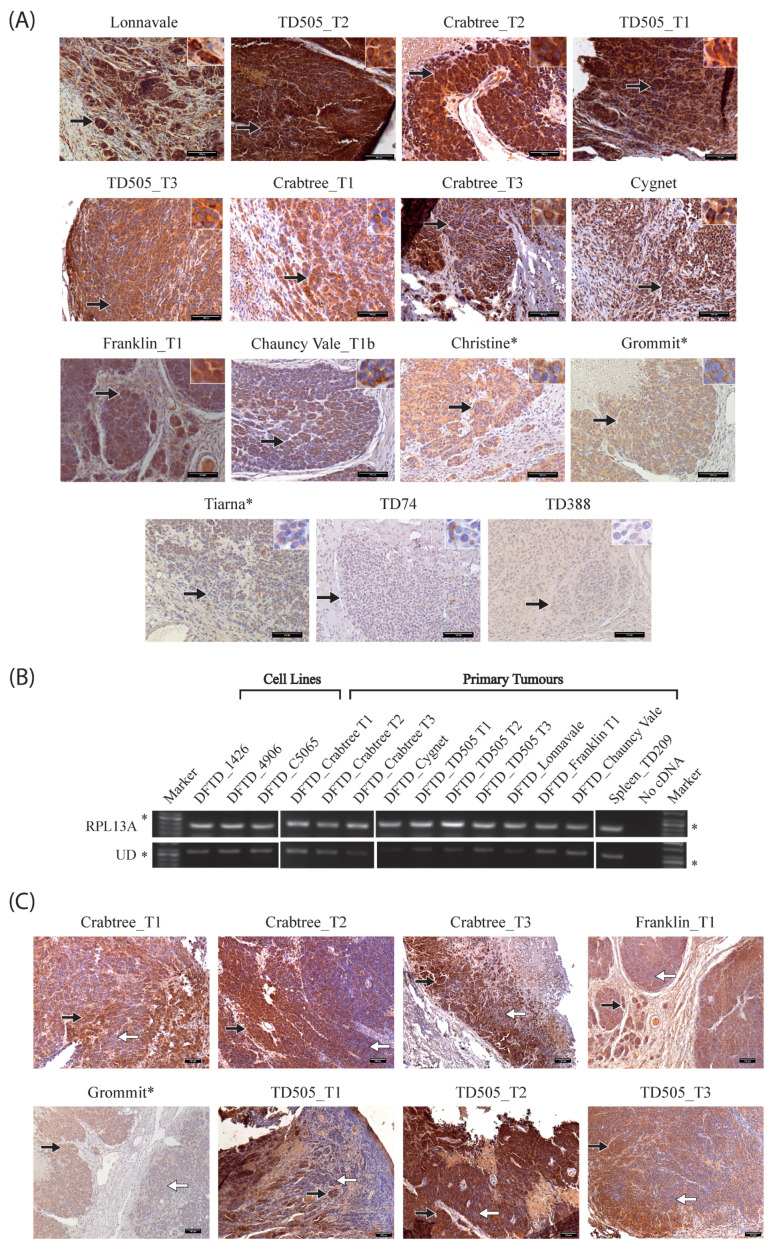
**Saha-UD expression varies between and within DFTD tumours.** (**A**) Immunohistochemistry (IHC) staining of formalin-fixed paraffin-embedded (FFPE) DFTD tumour samples using an anti-Saha-UD antibody. Black arrows indicate DFTD tumour cells with positive staining for the Saha-UD. Images were taken at 20× magnification. Positive cells are stained brown; nuclei are stained blue. Asterisks indicate tumours obtained from devils inoculated with cell line DFTD_C5065. Scale bars = 100 µm. (**B**) RT-PCR on the DFTD cell lines and primary tumours for *Saha-UD*. *RPL13A*, encoding a ribosomal protein, was used as a loading control. A no cDNA control is included for each. Asterisks indicate 250 bp on the DNA ladder. (**C**) IHC images for FFPE DFTD tumour samples showing heterogeneous intratumour staining for the Saha-UD. Black arrows indicate DFTD tumour cells with strong staining for the Saha-UD, and white arrows indicate DFTD tumour cells that are negative or have lower levels of staining for the Saha-UD. Images were taken at 10× magnification. Positive cells are stained brown; nuclei are stained blue. Asterisks indicate tumours obtained from devils inoculated with cell line DFTD_C5065. Scale bars = 100 µm.

To investigate the Saha-UD expression in DFTD, the UD(3) antibody was used to stain 15 DFTD tumour samples by IHC (Figure 2A). Across these samples, collected between May 2006–April 2015, Saha-UD was expressed in 13 samples, but there was varied Saha-UD expression, from very strong staining seen for Crabtree_T2, Lonnavale, and TD505_T2 to negative tumours TD74 and TD388. Thus, while Saha-UD is expressed in many DFTD tumours, it is not expressed in all tumours and is expressed at different levels. Among the tumours stained by IHC, there were two devils for which we had three tumours: Crabtree and TD505. Interestingly, for both animals, while all three tumours expressed Saha-UD, the strength of staining varied between the different tumours (Figure 2A).

To confirm the positive Saha-UD expression observed with the UD(3) antibody by IHC, 10 primary DFTD samples stored in RNAlater and taken from the same tumours stained by IHC, were tested by RT-PCR. Included in this analysis were three DFTD cell lines (DFTD_1426, DFTD_4906, and DFTD_C5065) and devil spleen (Spleen_TD209) as a positive control. Samples were amplified using primers specific for *Saha-UD* and a ribosomal protein, *RPL13A*, as a control (primers detailed in Table 2). Consistent with previous results, DFTD cell lines were positive for the *Saha-UD* heavy chain [30,32,33]. All DFTD primary tumours were positive by IHC and RT-PCR, confirming the results obtained with the UD(3) antibody. Tumours negative for the Saha-UD were not tested by RT-PCR as samples stored in RNAlater were not available. These results confirm the Saha-UD expression in DFTD tumours from different individuals, but it is difficult to compare expression levels as, based on the IHC results, some contamination from host cells is expected in the biopsies.

In addition to the variations in Saha-UD expression between the DFTD tumours, there was heterogeneity within tumours (Figure 2C). Heterogeneity was classed as samples that contained both tumour cells with high-moderate Saha-UD expression and tumour cells that were low-negative. Thirteen out of the 15 tumours showed intratumour heterogeneity, where strong/moderate Saha-UD-positive tumour cells neighbour tumour cells with weaker staining or no staining at all. For half of the heterogenous tumour samples, this was observed throughout the tumour, for example, Chauncy Vale_T1b (Figure 2A), TD505_T2, and TD505_T3 (Figure 2C). The other half had areas with consistent Saha-UD expression, either positive or negative, in addition to areas with interspersed tumour cells of varying Saha-UD expression, as observed in Crabtree_T1, Crabtree_T2, and Grommit (Figure 2C). Interestingly, there was no clear pattern for areas that were high expressing and areas that were low or negative. Five tumours had areas where there appeared to be a higher expression of the Saha-UD on the edges of a cluster of tumour cells. The only tumours showing consistent staining throughout the tumour were Christine and TD388, the latter being classed as Saha-UD negative.

### 2.3. Distinct Patterns of Staining for Nonclassical Saha-UD versus Classical (Saha-UA, -UB, & -UC)

**Figure 3:**
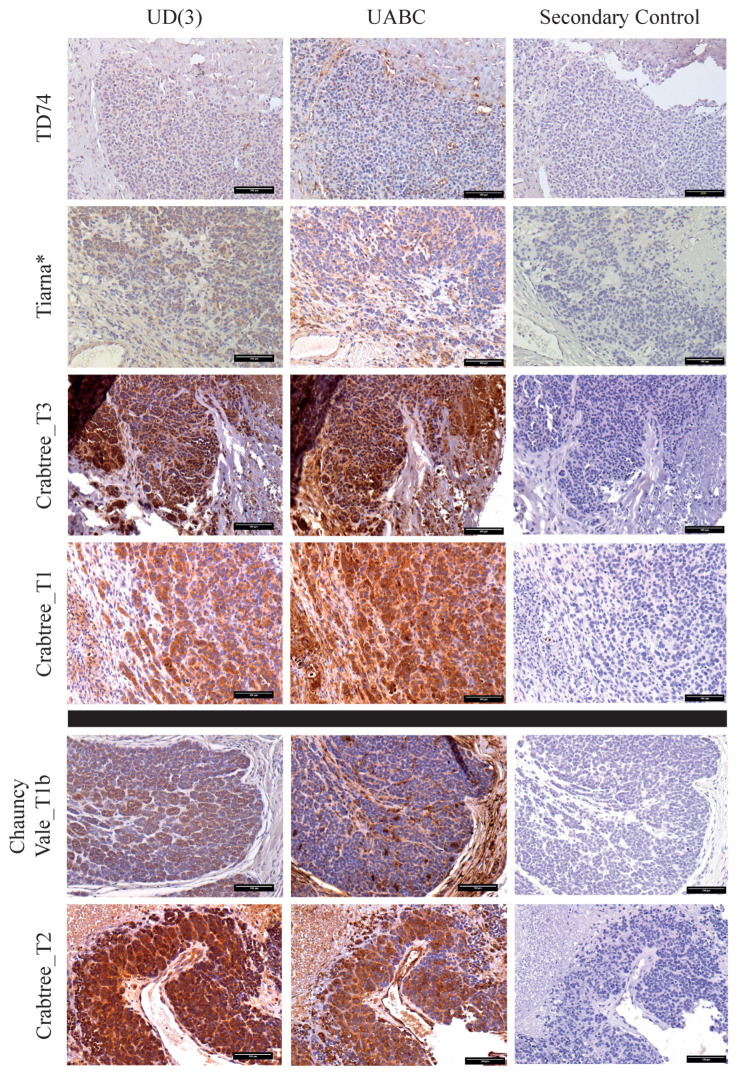
**Patterns of staining observed when comparing nonclassical Saha-UD versus classical MHC class I staining in DFTD.** Representative images of staining patterns observed for DFTD tumours when staining for classical MHC class I, Saha-UA, -UB, and -UC, with antibody α-UA/UB/UC_15-25-8 (UABC), and nonclassical MHC class I, Saha-UD, with antibody α-UD_14-37-3 (UD (3)). Images were taken at 20× magnification. Positive cells are stained brown; nuclei are stained blue. Asterisks indicate tumours obtained from devils inoculated with the cell line DFTD_C5065. Scale bars = 100 µm.

Based on our hypothesis that nonclassical Saha-UD is upregulated in DFTD tumours to prevent NK cell activation due to the ‘missing self’, we would expect higher levels of Saha-UD expression when there is lower classical MHC-I expression. To compare the expression of classical MHC-I, Saha-UA, -UB, and -UC with that of nonclassical Saha-UD, serial sections of the 15 DFTD tumours, previously stained using the UD(3) antibody, were stained for classical MHC-I expression using the pan-specific UABC antibody (α-UA/UB/UC_15-25-8) (Figure 3).

As with the nonclassical Saha-UD expression, there is variation in classical MHC-I expression between the DFTD tumours, with staining ranging from strong to negative. This contrasts previous findings that classical MHC-I transcription is low in cell lines and low or not expressed in DFTD primary tumours [19]. There was also intra-tumour heterogeneity for classical MHC-I expression, present in all 13 tumours that stained positively for classical MHC-I. Heterogeneity was observed throughout the tumours. In nine of these tumours, the heterogeneous classical MHC-I staining occurred in a similar pattern to the Saha-UD staining.

The inverse correlation we had expected between the Saha-UD and classical MHC-I expression was not present across all 15 tumours. However, there were two general expression patterns observed, examples of which are shown in Figure 3. The most common pattern, observed in 11 of the tumours, was that staining for the Saha-UD and classical MHC-I mirrored each other (Figure 3; top panel). For example, there is either strong staining for Saha-UD and classical MHC-I or staining for both is weak. While the expression of Saha-UD and classical MHC-I cannot be directly compared, as there may be differences in the binding affinity of the antibodies, it is interesting that most of the tumours had similar levels of staining by both antibodies. The two tumours classified as Saha-UD-negative, TD388 and TD74, were also negative for classical MHC-I.

The second pattern observed is that a subset of four tumours had stronger staining for Saha-UD than classical MHC-I (examples in Figure 3; bottom panel). These tumours had strong staining for the Saha-UD, with staining for classical MHC-I weaker or completely negative. Though this pattern more closely fit with our hypothesis, two of the four tumours did have moderate classical MHC-I expression.

## 3. Discussion

We have generated and validated a specific monoclonal antibody against the devil nonclassical Saha-UD (α-UD_14-37-3), which shows little cross-reactivity with other devil recombinant MHC-I proteins (classical Saha-UC and nonclassical Saha-UK). IHC shows that DFTD tumours express Saha-UD, though expression varies between tumours. Saha-UD expression in 10 of the DFTD samples was also confirmed by RT-PCR using Saha-UD specific primers. Interestingly, DFTD tumours are heterogeneous for Saha-UD expression, (Figure 2C) and, unexpectedly, we observe varied classical MHC-I expression between tumours.

The expression of the nonclassical Saha-UD heavy chain has previously been found in three DFTD cell lines [30,32,33], and this study provides the first evidence of Saha-UD expression in primary DFTD biopsies. Based on IHC staining, Saha-UD expression is common in DFTD tumours from different individuals. However, this analysis is restricted to 15 tumours over a limited geographic range. As it is possible that Saha-UD expression is changing over time and by location in DFTD, more samples are needed over a greater period to understand broader trends in Saha-UD expression and the evolution of DFTD immune escape.

**Figure 4:**
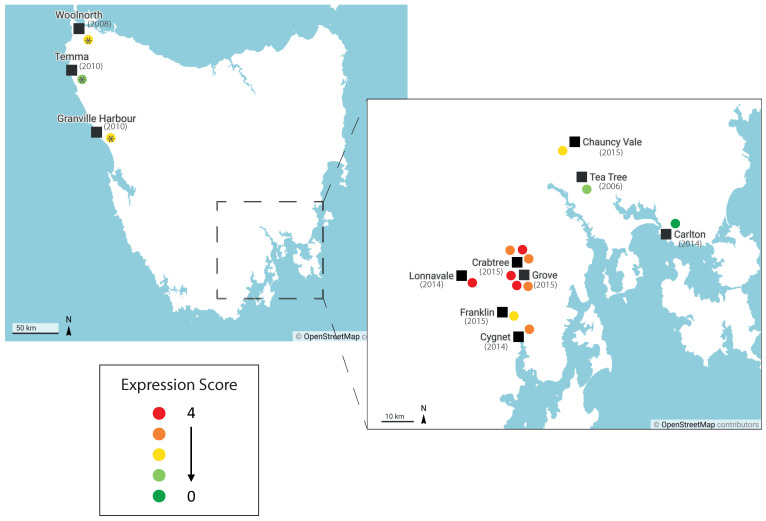
**Expression scores for nonclassical Saha-UD by DFTD sample location.** Map of Tasmania with sample locations for DFTD tumours. Each dot represents a single tumour; the colour indicates the expression score. Asterisks indicate tumours inoculated during vaccination [35] or transmission trials in captive devils obtained from these locations. Expression scores were assigned based on immunohistochemical staining of DFTD tumour samples. An expression score of 4 indicates strong staining; 0 indicates no staining. Expression score criteria are detailed in Figure S5. Map created using datawrapper.de.

Saha-UD expression by DFTD tumours may reflect the genetic evolution of the cancer as it has spread across Tasmania. The biopsy samples in this study are derived from two broad geographical areas in Tasmania. Devils captured from the Northwest of Tasmania were inoculated with the DFTD cell line C5065, obtained from Eastern Tasmania in 2005, and wild tumours were collected from locations in and around the d’Entrecasteaux peninsula, in the Southeast (See Figure S3 for a map showing sample locations and Table S1). Interestingly, tumours from the d’Entrecasteaux peninsula exhibited higher expression than both tumours to the East of this area and tumours from the inoculated devils (Figure 4). Though sample number is limiting, it is possible the variation in the Saha-UD expression is due to genetic differences between the DFTD tumours in these areas [34]. Recent genotyping of DFTD tumours has found that early in its clonal evolution, DFTD developed into multiple clades, some of which have persisted and become dominant in particular geographic areas [34]. Therefore, Saha-UD expression may be reflective of DFTD sublineages.

As it has been shown that dominant clades in a particular area can change over time [34], the date of the sample collection may be an important factor in the Saha-UD expression. While time in culture will have altered the C5065 cell line since it was collected in 2005, it is interesting that tumour samples from the inoculated devils match the natural tumour from 2006, TD74. Conversely, newer tumours appear to have a broader range of Saha-UD expression. Thus, Saha-UD variability may have increased with time, highlighting the importance of elucidating both spatial and temporal factors in Saha-UD expression, as the two may be interlinked.

The variation in Saha-UD expression may also be due to the individual interactions of tumours with the host immune system. The expression of Saha-UD is lower in Tiarna than in Grommit and Christine (Figure 2A), despite all being inoculated with the cell line C5065, and Grommit and Tiarna both show intratumour heterogeneity. Intratumour heterogeneity, observed in 13 of the 15 DFTD tumours, highlights that Saha-UD expression may be plastic in response to local tissue conditions, such as infiltrating immune cells. It has been shown that DFTD cells exhibit mesenchymal plasticity in response to immune activation following immunisation [36], changing to a dedifferentiated, immunosuppressive state. Though MHC-I expression was not investigated, it could be controlled by similar pathways. However, *Saha-UD* is located in a different region as compared to the classical MHC-I genes and is missing many upstream regulatory elements present for classical MHC-I, including an interferon stimulated response element [16]. Thus, its response to local inflammation is predicted to be limited.

If Saha-UD expression was solely dependent on the local immune environment, patterns of heterogeneity within the tumour would be expected. However, this was not seen in most tumours. Some tumours had small areas with slightly higher Saha-UD expression on the outside of clusters of tumour cells, which might be expected if Saha-UD was being upregulated in response to infiltrating immune cells, but this was not conclusive or present throughout the tumour. Thus, Saha-UD expression is likely to be associated with a combination of the sublineage of DFTD, the history of host devils encountered by the tumour cells, and the specific interactions with the current host devil immune system. This is highlighted by varying levels of Saha-UD expression in different DFTD tumours infecting the same devil (see Crabtree and TD505, Figure 2A). These tumours may originate from bite wounds from different devils and/or derive from an infection with different clades of DFTD [34]. These tumours may have been subjected to different selective pressures as they moved through the population prior to infection in the current devil, which may produce differential interactions with the host immune system. This is akin to the evolution observed in other emerging pathogens as they spread through a population: acquiring mutations and genetic changes in response to the pressures of the host environments they encounter, which leads to the development of variants.

The DFTD tumours examined are also heterogenous for classical MHC-I expression, which was unexpected given previous findings that DFTD cells are MHC-I negative [19]. This may be due to plasticity in the classical MHC-I expression, afforded by epigenetic mutations in genes for components of the peptide presentation pathway [21], which can be upregulated by cytokines (for example, IFN-γ). This would be a similar progression as observed in the transmissible cancer in dogs, canine transmissible venereal tumour (CTVT), which regulates the expression of MHC-I during an infection of a single host, with MHC-I downregulated during its progressive growth phase and upregulated in response to cytokines during the regressive phase [37,38]. An alternative scenario is that subclonal lineages of DFTD are emerging that express classical MHC-I. MHC-I expression on DFTD cells has been shown to elicit an immune response in devils [39]. Therefore, emergence of MHC-I-positive tumours may contribute to recent findings of devil immune responses [18] and tolerance to DFTD [11].

Based on our original hypothesis, the loss of classical MHC-I would be expected to correlate with the gain of Saha-UD expression to mitigate the effects of NK cells. Expression levels of Saha-UD and classical MHC-I cannot be directly compared using IHC as antibody affinity may differ. However, all tumours that expressed classical MHC-I also expressed nonclassical Saha-UD. In nine of the 13 tumours that were heterogeneous for classical MHC-I expression, the areas of heterogeneity match between Saha-UD and classical MHC-I. Thus, it appears that the expression of nonclassical Saha-UD and classical MHC-I are linked, potentially due to genes associated with the antigen presentation pathway. Though these results do not support our hypothesis, nonclassical Saha-UD expression may still play a role in immune evasion of DFTD. Given that classical MHC-I expression in DFTD is antigenic [39] and that Saha-UD is expressed when there is classical MHC-I, nonclassical Saha-UD could be creating an immunosuppressive environment to prevent host recognition. In humans, the expression of nonclassical HLA-E, HLA-F, and HLA-G by extravillous trophoblasts induces maternal immunotolerance of foetal cells, preventing recognition of paternal alloantigens via the classical HLA-C molecules [40,41]. We postulate that Saha-UD binds peptides, due to the similarity to classical MHC-I sequences [16,27], and is expressed at the cell surface as it binds β_2_m [30]. Therefore, so it is possible that Saha-UD is providing an immunosuppressive signal to the immune system. However, to investigate potential roles for Saha-UD in immune evasion, further work is needed to confirm its ligand binding properties and interactions with immune cells. In addition, a wider range of DFTD samples will be tested for Saha-UD expression to investigate whether there are differences over time, by location, or by DFTD clades.

## 4. Materials and Methods

### 4.1. Animals

Wild Tasmanian devils were trapped or found dead from road trauma or other causes. Tissue biopsies and fine needle aspirates were collected postmortem or from live devils, which were subsequently released. Samples collected were formalin fixed (10% neutral buffered formalin) and paraffin embedded or were stored in RNAlater.

Two tumour samples were from devils, Grommit and Tiarna, who were immunised with an irradiated cell line DFTD_C5065 (protocol detailed in [35]). C5065 (RRID:CVCL_LB79) is a cell line established from a DFTD biopsy in 2005. Despite irradiation of the cell line, tumours grew at the site of inoculation. Christine was inoculated with DFTD_C5065 as part of an unrelated transmission trial.

All animal procedures were performed under a standard operating procedure approved by the general manager through the Natural and Cultural Heritage Division, Tasmanian Government Department of Primary Industries, Parks, Water and the Environment, or under University of Tasmania Animal Ethics Committee Permit A0014976. Information for the devil samples used in this paper are listed in Table S1, and sample collection locations are shown in Figure S3.

### 4.2. Sequence Analysis

To calculate the percentage sequence identity for the alpha 1–alpha 2 domain amino acid sequences of devil MHC-I genes (Table 1), the sequences were aligned in CLC Main Workbench 22 and the percentage sequence identity calculated in Bioedit using the sequence identity matrix function. Sequence identity was compared between the classical MHC-I (devil MHC-I reference sequence SahaI-01; NCBI: NP_001267784.1), nonclassical Saha-UD (SahaI*12; NCBI: NP_001267783.1), nonclassical Saha-UK (GenBank: AIS75088.1), and nonclassical Saha-UM (GenBank: AIS75089.1).

The model of the devil nonclassical Saha-UD (Figure 1A) was generated using an AlphaFold Colab notebook created by DeepMind [42]. Saha-UD is modelled on the alpha 1–alpha 3 domain of the amino acid sequence (SahaI*12; NCBI: NP_001267783.1).

### 4.3. Anti-Saha-UD Antibody Generation

Antibodies against the Tasmanian devil MHC-I molecules have previously been designed and validated in our lab [32]. These were against the nonclassical Saha-UK, using peptide sequence ‘RITHRTHPDGKVTL’ from the alpha-3 domain, and a pan-specific antibody for the classical Saha-UA, -UB, and -UC, against the peptide sequence ‘WMEKVQDVDPGYWE’ from the alpha-1 domain (Figure 1A).

For the generation of an antibody against the nonclassical Saha-UD, MHC-I transcripts representing all known devil MHC-I alleles [31] were aligned and translated into protein sequences using CLC workbench [32]. The alpha 1 domain was manually assessed for regions of low amino acid identity between Saha-UD alleles, classical MHC-I (Saha-UA, -UB, and -UC) and Saha-UK (Figure 2A). Using this sequence, the peptide ‘WIEKMENVDRDYWE’ was synthesised for immunisations against Saha-UD. Mice were immunised subcutaneously twice with a mixture of GERBU adjuvant and 50 μg of WIEKMENVDRDYWE-C coupled to diphtheria toxoid via the N-terminal cysteine. The final boost was performed by intravenous injection, without GERBU, using 25 ug of antigen. Three days later, spleen lymphocytes were fused with the SP2 cell line using PEG as a fusogen. Hybridomas were selected based on reactivity in the ELISA against both N- and C-terminal-coupled peptide. Antibodies were subsequently screened against Tasmanian devil spleen samples and verified against recombinant expressed MHC-I by Western blot, as described below.

### 4.4. Recombinant MHC Class I Proteins

Recombinant devil MHC-I heavy chain proteins were used to determine the specificity of anti-Saha-UD antibodies, specifically Saha-UC (ensembl:ENSSHAG00000000117; Devil_ref v7.0), Saha-UK (ensembl:ENSSHAG00000002942; Devil_ref v7.0), and Saha-UD (ensembl: ENSSHAG00000010776; Devil_ref v7.0). The MHC-I classical Saha-UC and nonclassical Saha-UK full length recombinant proteins were previously generated in our lab [32]. For this work, we also generated recombinant protein for the nonclassical Saha-UD using the same method. Briefly, exons 2–4 from Saha-UD were amplified from the full gene sequence (Ensembl identifier: ENSSHAG00000010776.2) using primers Saha-138 and Saha-48 (Table 2). The subsequent amplicons were cloned into the pET22b^+^ vector (Novagen) and transformed into competent dh5α E. coli cells. Clones from single colonies were grown in LB with ampicillin for 12 h, the plasmid was isolated using the NucleoSpin Plasmid kit (Macherey-Nagel, Düren, Germany), and the transcripts were sequenced in both directions. pET22b^+^-SahaUD plasmids with the correct sequence were transformed into Rosetta pLysS cells (Novagen). Bacterial colonies containing the plasmid were cultured to OD_600_ 0.6, and protein expression was induced with 1mM Isopropyl β-D-1-thiogalactopyranoside (IPTG). The IPTG-induced cell suspension was spun down at 13,000 rpm for 1 min and resuspended in a 25 µL denaturing loading buffer (500 μL 2X Lamelli sample buffer, 50 μL β-mercaptoethanol, and 450 μL dd-H_2_O) for direct analysis by Western blot (see below).

### 4.5. SDS-PAGE and Western Blot

To test the specificity of anti-Saha-UD antibodies, the recombinant Saha-UC and Saha-UK protein (described above) was run on a Western blot. 6 µg of Saha-UC or Saha-UK solubilised recombinant protein was made up to a total volume of 25 µL with a loading buffer (500 μL 2X Lamelli sample buffer, 50 μL β-mercaptoethanol, and 450 μL dd-H_2_O). The Saha-UD samples described above were run alongside the Saha-UC and Saha-UK. Preparations were heated to 95 °C for 10 min, and 20 µL were added per well.

Samples were run on a 12% acrylamide gel (National Diagnostics ProtoGel^®^ 30%) using Laemmli buffers and the Fisherbrand™ Vertical Gel Tank. Proteins were transferred to a nitrocellulose blotting membrane (Amersham Protran GE Healthcare Life Sciences, Uppsala, Sweden) in a transfer buffer (25 mM Tris, 190 mM glycine, and 20% (*v*/*v*) methanol) using a vertical gel tank (Geneflow). The membrane was blocked for 1 hr with 5% milk in TBST (150 mM NaCl, 0.1% (*v*/*v*) Tween20, 5% (*w*/*v*) milk powder, 50 mM TrisCl (pH 8)); it was then washed in TBST (150 mM NaCl, 0.1% (*v*/*v*) Tween20, 50 mM TrisCl (pH 8)), and incubated with a neat hybridoma supernatant at 4 °C overnight. Membranes were washed with TBST, incubated with a secondary antibody (IRDye^®^ 680RD Goat anti-Mouse IgG (H + L)) in 5% milk in TBST for 1 hr at room temperature, and washed in TBST. Membranes were visualised using the LI-COR Odyssey^®^ scanner.

### 4.6. Immunohistochemistry (IHC)

Spleen and DFTD tissue samples were fixed in a 10% (*v*/*v*) PBS-buffered formalin solution for 2 to 4 days before being processed and embedded in paraffin blocks. The samples were cut using a microtome at a thickness between 4−10 µm and mounted onto coated slides.

Sections were deparaffinized in xylene and rehydrated through graded alcohol. Antigen retrieval was performed by a water bath (95 °C) in a citrate buffer solution (10 mM citric acid, 0.05% Tween20, pH 6) for 40 min followed by cooling for 15 min. Endogenous peroxidase was blocked by the incubation of slides with 3% H_2_O_2_ (Sigma Aldrich, St. Louis, MI, USA) for 10 min, and the nonspecific protein binding was blocked with a 10% (*v*/*v*) goat serum in PBS for 30 min. Sections were incubated with primary antibodies (listed in Table S2) at 4 °C overnight. A peroxidase-coupled secondary antibody (EnVision Peroxidase/DAB+ kit; Dako) was used to detect primary antibody binding; incubating sections with HRP for 30 min and colour developed with DAB chromogen for 5 min. Sections were counterstained with haematoxylin (Vector hematoxylin nuclear counterstain (Gill’s Formula)) for 4 min, differentiated in 2% (*v*/*v*) acetic acid, and blued in 0.2% (*v*/*v*) ammoniated water. Sections were dehydrated through graded alcohol, transferred to xylene, and cover-slipped (using National Diagnostics Omnimount Histological Mounting Medium). Images were captured using the Nikon Eclipse 400 microscope, Retiga 2000R camera, and Q-capture pro 7 computer software. Scale bars were added to images using ImageJ software.

### 4.7. RT-PCR

The NucleoSpin RNA kit (Macherey-Nagel, Düren, Germany) was used to extract RNA from cells lines and TRI Reagent (Sigma-Aldrich) used for tissue samples, both according to the manufacturer’s instructions. 1 μg of RNA was reverse transcribed to cDNA using the RevertAid First Strand cDNA Synthesis kit (Thermo Fisher, Waltham, MA, USA), according to the manufacturer’s instructions.

Dev Men UD primers [32] were used to amplify the nonclassical MHC-I, *Saha-UD* by PCR. RPL13A primers [19], amplifying a ribosomal protein, were used as a control. Primers and annealing temperatures are listed in Table 2. 500 ng of cDNA was amplified using Phusion High-Fidelity DNA Polymerase (Thermo Fisher), according to the manufacturer’s instructions (see Table 3 for PCR reaction conditions) and appropriate negative controls. PCR products were run on a 1.2% (*w*/*v*) agarose gel with GelRed and visualised using the SynGene P*Xi* machine and SynGene software.

## Figures and Tables

**Table 1:** Percentage sequence identity of the alpha 1–alpha 2 domain amino acid sequence of devil MHC class I genes: classical MHC class I (SahaI-01) and nonclassical Saha-UD (SahaI*12), Saha-UK, and Saha-UM.

MHC Class I	Classical (SahaI-01)	Saha-UD (SahaI*12)	Saha-UK	Saha-UM
**Classical** **(SahaI-01)**		78.2%	60.6%	52.1%
**Saha-UD** (SahaI*12)	78.2%		58.1%	52.4%
**Saha-UK**	60.6%	58.1%		43.1%
**Saha-UM**	52.1%	52.4%	43.1%	

**Table 2:** Primer sets.

Gene Amplified	Primer Names	Primer Sequence (5′ -> 3′)	Product Length (bp)	Annealing Temperature (°C)
Nonclassical MHC Class I *Saha-UD* (exon 2–4)	Saha138	GCCATATGGGCTCTCACTCCTTGAGGTATTTCGGCACCAC	1044	63
	Saha48	ATGCAAGCTTGGCTTTGGCTGTCAGAGAGACATCTGACC		
*RPL13A*	Saha120	ACAAGACCAAGCGAGGCCAGG	300	60
	Saha121	GCCTGGTATTTCCAGCCAACCTCA		
Nonclassical MHC Class I *Saha-UD*	Dev Men UD (F)	ATGGAGAATGTGGACCGGGAC	275	60
	Dev Men UD (R)	TGAGTTCACTGCCTCATTCACT		

**Table 3:** PCR reagents and conditions.

PCR Reagents		RCR Reaction Conditions			
Reagent	Final Concentration	Cycle Element	Temperature (°C)	Time (s)	Number of Cycles
cDNA	500 ng	Initial denaturation	98	30	1
DNA polymerase (Thermo Fisher *Phusion*)	0.5 U	Denaturation	98	10	30
Primers	0.6 µM	Annealing	Temperatures for primers in Table 2	30	
dNTPs	200 µM	Elongation	72	30	
Phusion High Fidelity buffer	1X	Final Elongation	72	300	1
ddH_2_O	To total volume of 25 µl				

## Data Availability

The data presented in this study are available in Hussey et al., 2022 and associated Supplementary Material.

## Supplementary Materials

The following supporting information can be downloaded at: https://www.mdpi.com/article/10.3390/pathogens11030351/s1, Figure S1: Screening of nonclassical anti-Saha-UD antibodies by immunohistochemistry using devil spleen samples; Figure S2: Recombinant heavy chain proteins for classical Saha-UC and nonclassical Saha-UK, run on an SDS-PAGE gel stained with Coomassie Blue; Figure S3: Map of sample locations for Devil Facial Tumour Disease (DFTD) samples; Figure S4: Immunohistochemistry staining of Devil Facial Tumour Disease (DFTD) samples for periaxin expression [43,44]; Figure S5: Expression score criteria and mapped DFTD classical MHC class I expression scores; Table S1: Details of Devil Facial Tumour Disease (DFTD) samples used in this paper; Table S2: Primary antibodies.

## Abbreviations

β_2_m	β_2_-microglobulin
CTVT	Canine Transmissible Venereal Tumour
DFTD	Devil Facial Tumour Disease 1
FFPE	Formalin-Fixed Paraffin-Embedded
HLA	Human Leukocyte Antigen
IFN-γ	Interferon-γ
IHC	Immunohistochemistry
NK Cell	Natural Killer Cell
MHC-I	Major Histocompatibility Complex Class I
PBS	Phosphate Buffered Saline
PCR	Polymerase Chain Reaction
PRC2	Polycomb Repressive Complex 2
TAP	Transporter Associated with Antigen Processing
