# Supplementary material for: Expression of the Nonclassical MHC Class I, Saha-UD in the Transmissible Cancer Devil Facial Tumour Disease (DFTD)
Source: Pathogens. 2022 Mar 14;11(3):351. doi: 10.3390/pathogens11030351 (PMC8953681; doi:10.3390/pathogens11030351)
Supplement: Supplementary file 1 [file pathogens-11-00351-s001.zip › Hussey et al_Figure S1.pdf]

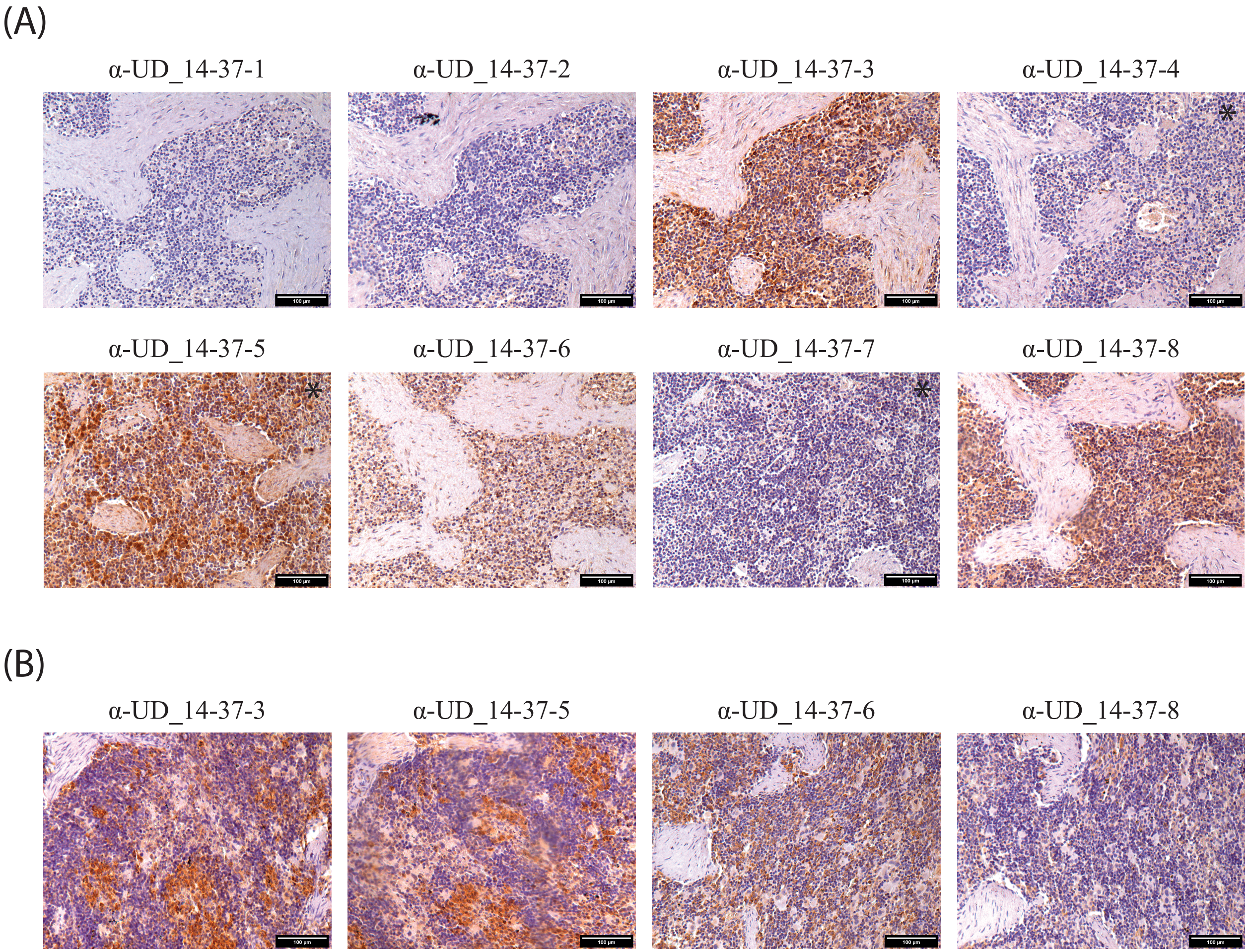

Figure S1. Screening of non-classical anti-Saha-UD antibodies by immunohistochemistry using devil spleen samples. 8 monoclonal antibodies against devil non-classical Saha-UD were generated by immunising mice using the peptide sequence 'WIEKMEN-VDRDYWE' **(A)** All 8 antibodies were screened by immunohistochemistry using devil spleen samples. Asterisks indicate images that are not serial. The image for antibody clone α-UD_14-37-4 was taken of a different area of the same tissue. α-UD_14-37-5 and α-UD_14-37-7 were tested on a different spleen sample to the other 6 antibodies. **(B)** The 4 antibodies that stained for spleen were tested again on serial sections of the same spleen sample. Due to quality of the tissue sample, serial images could not be taken for all 4 antibodies. Images for α-UD_14-37-3 and α-UD_14-37-5 are taken in the same area of tissue as each other, while α-UD_14-37-6 and α-UD_14-37-8 were taken of the same area. Images taken at 20x magnification. Positive cells are stained brown, nuclei are stained blue. Scale bars = 100 µm.
