# Supplementary material for: Expression of the Nonclassical MHC Class I, Saha-UD in the Transmissible Cancer Devil Facial Tumour Disease (DFTD)
Source: Pathogens. 2022 Mar 14;11(3):351. doi: 10.3390/pathogens11030351 (PMC8953681; doi:10.3390/pathogens11030351)
Supplement: Supplementary file 1 [file pathogens-11-00351-s001.zip › Hussey et al_Figure S3.pdf]

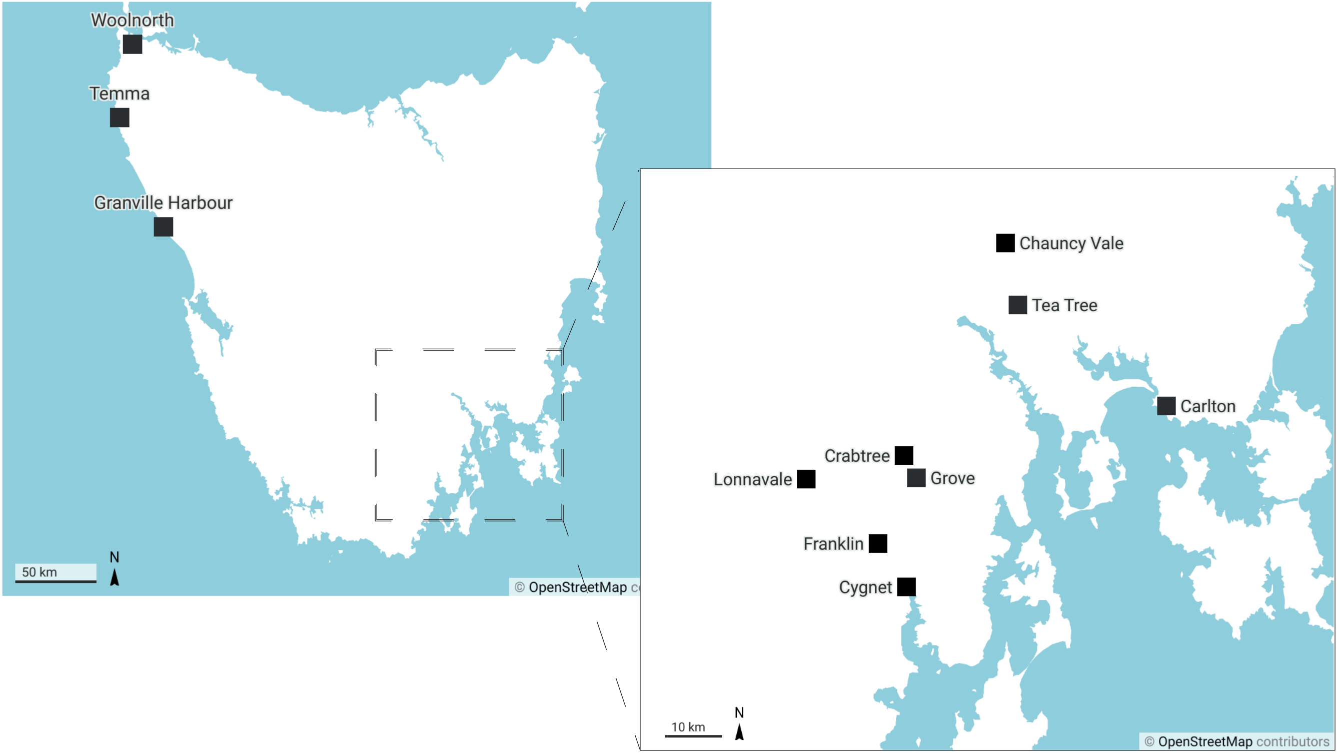

Figure S3. Map of sample locations for Devil Facial Tumour Disease (DFTD) samples. Map of Tasmania showing trapping locations for DFTD samples used in this paper. Map created using datawrapper.de.
