# Supplementary material for: Expression of the Nonclassical MHC Class I, Saha-UD in the Transmissible Cancer Devil Facial Tumour Disease (DFTD)
Source: Pathogens. 2022 Mar 14;11(3):351. doi: 10.3390/pathogens11030351 (PMC8953681; doi:10.3390/pathogens11030351)
Supplement: Supplementary file 1 [file pathogens-11-00351-s001.zip › Hussey et al_Figure S4.pdf]

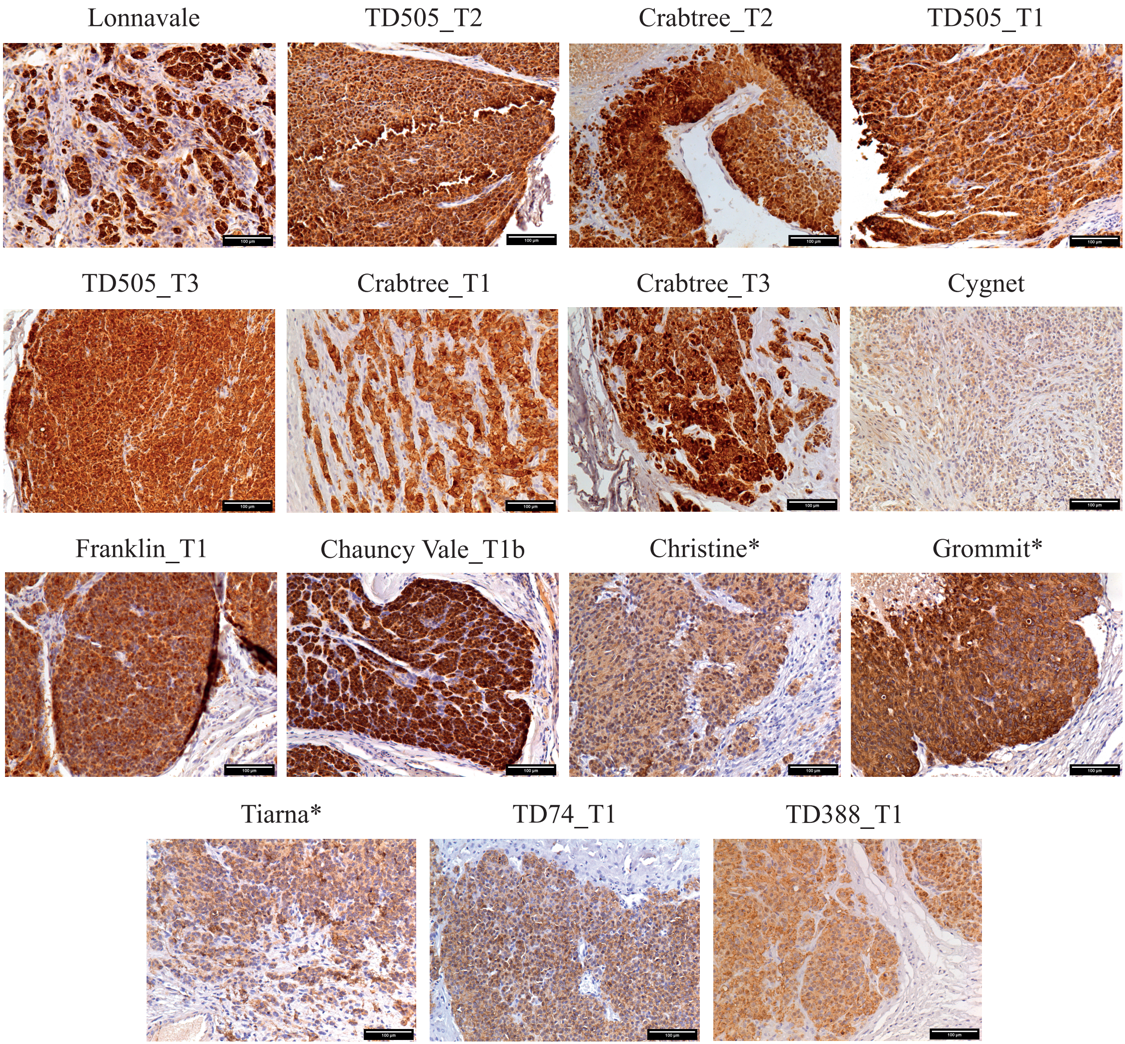

Figure S4. Immunohistochemistry staining of Devil Facial Tumour Disease (DFTD) samples for periaxin expression. Periaxin is a myelinating protein, used as a specific marker for DFTD cells [43,44]. Images taken at 20x magnification. Positive cells are stained brown, nuclei are stained blue. Asterisks indicate tumours obtained from devils inoculated with cell line DFTD_C5065. Scale bars = 100 μm.
