# Supplementary material for: Expression of the Nonclassical MHC Class I, Saha-UD in the Transmissible Cancer Devil Facial Tumour Disease (DFTD)
Source: Pathogens. 2022 Mar 14;11(3):351. doi: 10.3390/pathogens11030351 (PMC8953681; doi:10.3390/pathogens11030351)
Supplement: Supplementary file 1 [file pathogens-11-00351-s001.zip › Hussey et al_Figure S5.pdf]

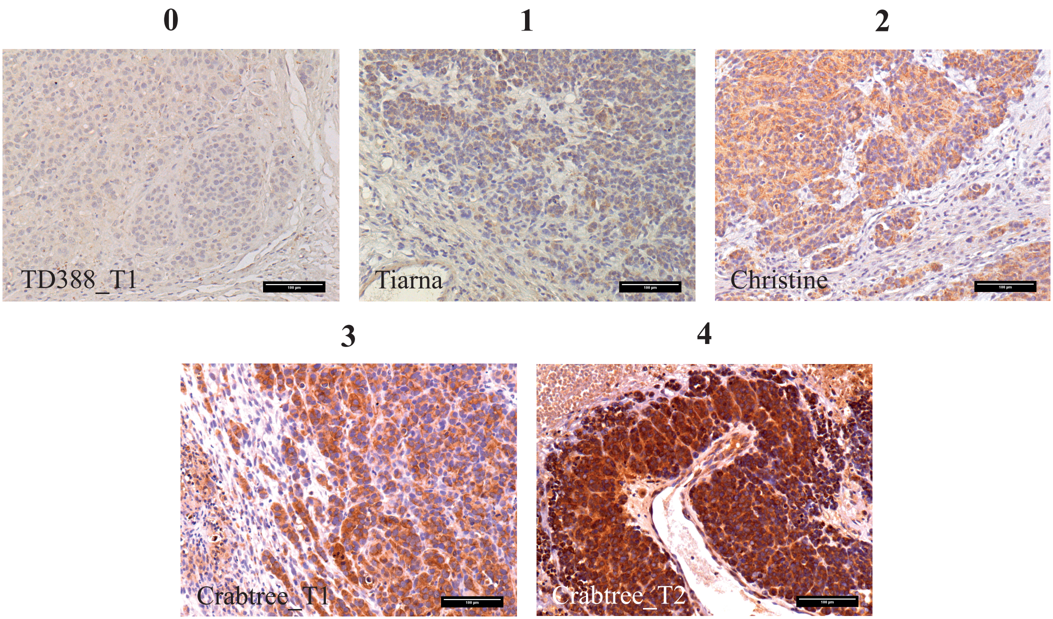

Expression Score	Strength of Staining	Cytosolic/Membrane
4	Very Strong	Mostly Membrane
3	Strong	Cytosolic and Membrane
2	Moderate	Cytosolic and Membrane
1	Weak	Mostly Cytosolic
0	No Staining	-

Figure S5. Expression score criteria and mapped DFTD classical MHC class I expression scores. Table detailing expression score criteria for tumours stained by immunohistochemistry. Example images are shown for each expression score, using DFTD tumours stained for non-classical Saha-UD (also presented in **Figure 2A**). Images taken at 20x magnification. Positive cells are stained brown, nuclei are stained blue. Scale bars = 100 µm.
