# Supplementary material for: Expression of the Nonclassical MHC Class I, Saha-UD in the Transmissible Cancer Devil Facial Tumour Disease (DFTD)
Source: Pathogens. 2022 Mar 14;11(3):351. doi: 10.3390/pathogens11030351 (PMC8953681; doi:10.3390/pathogens11030351)
Supplement: Supplementary file 1 [file pathogens-11-00351-s001.zip › Hussey et al_Table S1.pdf]

Table S1. Details of Devil Facial Tumour Disease (DFTD) samples used in this paper. Including the Tasmanian devil the sample was taken from, tissue samples, date collected, and the location where the sample was taken.

Devil Name	Microchip Number	Tissue Sample	Tumour Type	Date Collected	Location
Chauncy Vale		Tumour 1b	DFTD	March 2015	Chauncy Vale
Christine (TD111)		Tumour	DFTD	November 2008	Woolnorth
Crabtree		Tumour 1	DFTD	March 2015	Crabtree
Crabtree		Tumour 2	DFTD	March 2015	Crabtree
Crabtree		Tumour 3	DFTD	March 2015	Crabtree
Cygnet		Tumour	DFTD	March 2014	Cygnet
Franklin	N/a	Tumour 1	DFTD	April 2015	Franklin
Grommit (TD184)	982 009 105 175 123	Tumour	DFTD	June 2010	Granville Harbour
Lonnavale		Tumour	DFTD	July 2014	Lonnavale
TD74	N/a	Tumour 1	DFTD	May 2006	Tea Tree
TD74	N/a	Lymph Node	-	May 2006	Tea Tree
TD388		Tumour 1	DFTD	November 2014	Carlton
TD505	982000356444429	Tumour 1	DFTD	February 2015	Grove
TD505	982000356444429	Tumour 2	DFTD	February 2015	Grove
TD505	982000356444429	Tumour 3	DFTD	February 2015	Grove
Tiarna (TD182)	982 009 105 183 213	Tumour	DFTD	July 2010	Temma
