# Supplementary material for: Expression of the Nonclassical MHC Class I, Saha-UD in the Transmissible Cancer Devil Facial Tumour Disease (DFTD)
Source: Pathogens. 2022 Mar 14;11(3):351. doi: 10.3390/pathogens11030351 (PMC8953681; doi:10.3390/pathogens11030351)
Supplement: Supplementary file 1 [file pathogens-11-00351-s001.zip › Hussey et al_Table S2.pdf]

Table S2. Primary antibodies.

Antibody	Referred to as in this paper	Clone/Catalogue number	Dilution used	Reference/ Supplier
Non-classical MHC class I Saha-UD (1)		α-UD_14-37-1	Neat supernatant	This paper
Non-classical MHC class I Saha-UD (2)		α-UD_14-37-2	Neat supernatant	This paper
Non-classical MHC class I Saha-UD (3)	UD(3)	α-UD_14-37-3	Neat supernatant	This paper
Non-classical MHC class I Saha-UD (4)		α-UD_14-37-4	Neat supernatant	This paper
Non-classical MHC class I Saha-UD (5)	UD(5)	α-UD_14-37-5	Neat supernatant	This paper
Non-classical MHC class I Saha-UD (6)		α-UD_14-37-6	Neat supernatant	This paper
Non-classical MHC class I Saha-UD (7)		α-UD_14-37-7	Neat supernatant	This paper
Non-classical MHC class I Saha-UD (8)		α-UD_14-37-8	Neat supernatant	This paper
Classical MHC class I Saha-UA, -UB and -UC	UABC	α-UA/UB/UC_15-25-8	Neat supernatant	Caldwell et al. 2018 [32]
Non-classical MHC class I Saha-UK	UK	α-UK_15-29-1	Neat supernatant	Caldwell et al. 2018 [32]
Mouse IgG2a kappa isotype control (eBM2a)	IgG2a	14-4724-82	1:500	Invitrogen (ThermoFisher)
Periaxin		HPA001868	1:300	Sigma-Aldrich
